# Burden and Outcomes of Viral Respiratory Infections Among Adults in Bahrain: A Retrospective Cohort Study

**DOI:** 10.7759/cureus.106249

**Published:** 2026-04-01

**Authors:** Safa Alkhawaja, Rommel Acunin, Thuraya Zaid, Afaf Mohamed, Mohamed Alsaad, Mohamed Makhlooq, Muneer Mahdi, Alaa M Alzamrooni, Athraa S Naser, Nermin Saeed

**Affiliations:** 1 Medicine, Government Hospitals, Manama, BHR; 2 Infection Control, Government Hospitals, Manama, BHR; 3 Internal Medicine/Respiratory Unit, Government Hospitals, Manama, BHR; 4 Public Health, Ministry of Health - Bahrain, Manama, BHR; 5 Internal Medicine, Frimley Park Hospital, Frimley, GBR; 6 Internal Medicine, Arabian Gulf University, Manama, BHR; 7 Internal Medicine, Government Hospitals, Manama, BHR; 8 Pathology, Government Hospitals, Manama, BHR

**Keywords:** bahrain, icu admission, influenza, pneumonia, respiratory infections, rsv, sars-cov-2

## Abstract

Background

Viral respiratory infections remain a major cause of morbidity and mortality among adults, particularly those with chronic comorbidities. In Bahrain, most available evidence is derived from surveillance-based datasets that are limited by underreporting and incomplete clinical information. This study provides a comprehensive assessment of the burden, testing patterns, and outcomes of severe acute respiratory syndrome coronavirus-2 (SARS-CoV-2), influenza, and respiratory syncytial virus (RSV) among hospitalized adults using detailed clinical data from a major tertiary hospital.

Methods

A retrospective cohort study was conducted among patients aged ≥14 years admitted to Salmaniya Medical Center, Bahrain, with acute respiratory illness during the 2023-2024 winter season. Demographics, comorbidities, admission diagnoses, viral testing results, antiviral use, intensive care unit (ICU) admission, mechanical ventilation, and mortality were extracted from electronic medical records. Descriptive statistics, chi-square/Fisher's exact tests, multivariate logistic regression, and Kaplan-Meier survival analysis with Cox proportional regression model were performed.

Results

Among 943 hospitalized adults, 482 (51.1%) were aged >60 years, and 681 (72.2%) were non-Bahrainis. Of all patients, only 625 (66.3%) underwent viral testing, reflecting non-standardized testing practices. SARS-CoV-2 positivity was 5.1% (32/625), influenza positivity 6% (10/162), and RSV positivity 5.2% (3/57). Chi-square test revealed that SARS-CoV-2 infection was significantly associated with female gender (p = 0.008), age >60 years (p = 0.045), diabetes (p = 0.009), and higher mortality (21.9% vs. 10.1%; p = 0.036). The multivariate regression analysis showed that being female (AOR 2.78, CI: 1.25-6.19, p = 0.012) and having diabetes (AOR 2.54, CI: 1.13-5.70, p = 0.024) were independent predictors of SARS-CoV-2 infection. Kaplan-Meier curves demonstrated that SARS-CoV-2-positive patients had significantly shorter time to discharge than SARS-CoV-2-negative patients (p = 0.016). Influenza infection was associated with chronic lung disease (p = 0.045) and bronchial asthma (p = 0.023), with no deaths or ICU admissions among influenza‑positive cases. A small sample size (n = 3) limited RSV findings.

Conclusion

SARS-CoV-2 infection was relatively uncommon but was associated with markedly worse clinical outcomes, including higher in-hospital mortality and shorter survival. Influenza and RSV positivity rates were higher but substantially under-tested, limiting accurate burden estimation. These findings highlight the need for standardized three-virus testing panels, earlier antiviral initiation, and targeted vaccination strategies for high-risk adults, particularly those with diabetes and chronic lung disease.

## Introduction

Viral respiratory tract infections remain a major global cause of morbidity, mortality, and healthcare utilization, particularly among older adults and individuals with chronic medical conditions. The coronavirus disease 2019 (COVID-19) pandemic brought renewed global focus on respiratory pathogens and demonstrated that high-risk groups are particularly affected [1]. In addition to severe acute respiratory syndrome coronavirus-2 (SARS-CoV-2), respiratory syncytial virus (RSV) continues to play a major role in causing acute respiratory tract infections and severe acute respiratory illness (SARI). These illnesses often lead to hospital stays and worse outcomes for adults with other health problems [2-4]. Even though these infections are clinically important, their true impact in many regions, including the Middle East, remains unclear. This is mainly because of differences in testing, limited surveillance, and inconsistent reporting.

In Bahrain, most available evidence on respiratory viral infections originates from national surveillance systems. These systems provide valuable epidemiologic insights, but their usefulness is limited by underreporting, missing clinical data, and the risk of diagnostic misclassification [5,6].

Respiratory viruses remain among the most common causes of acute respiratory illness (ARI) worldwide, with significant clinical and economic consequences. The Global Burden of Disease (GBD) 2023 analysis shows that chronic respiratory diseases continue to have a major impact worldwide. In 2023, there were about 468.3 million cases and 4.4 million deaths from these conditions, making them a leading cause of illness and death. While the age-standardized mortality rate has dropped by 36.7% since 1990, the burden has shifted. Interstitial lung disease and pulmonary sarcoidosis have become more common, especially among older adults. The COVID-19 pandemic also affected these trends, causing a slight increase in new cases and slowing the previous decline in deaths. The main risk factors remain the same around the world. Smoking is still the biggest cause of COPD-related deaths, while high body mass index and silica exposure are major factors for asthma and pneumoconiosis [7]. SARS-CoV-2 continues to result in significant morbidity among older adults and individuals with chronic diseases during the post-pandemic period, as studies indicate ongoing risks of hospitalization and mortality in high-risk populations [1].

Influenza continues to be a major contributor to seasonal respiratory illness globally, resulting in millions of severe cases and hundreds of thousands of deaths annually [2,3]. Patients with chronic lung disease, asthma, heart disease, or diabetes have a higher risk of serious complications from the flu, such as pneumonia, respiratory failure, and death [8].

RSV was once thought to mainly affect children, but it is now known to cause serious respiratory illness in adults as well. Recent studies show that adults aged 65 and older are hospitalized with RSV at rates between 136.9 and 255.6 per 100,000, and the rates are even higher for those 75 and older [9-10]. In adults, RSV can resemble influenza or SARS-CoV-2, with symptoms ranging from mild upper respiratory tract infections to severe lower respiratory disease that may require intensive care [11]. Adults diagnosed with chronic obstructive pulmonary disease (COPD), asthma, cardiac disease, or immunocompromising conditions are especially susceptible. RSV is a recognized trigger for exacerbations of chronic lung disease [12,13]. Among hospitalized adults, 10% to 31% require intensive care and 3% to 17% require mechanical ventilation [12]. A recent multicenter study of 5,784 adults aged ≥60 years found that RSV was associated with higher intensive care unit (ICU) admission rates and greater risk of invasive mechanical ventilation or death compared with influenza or COVID-19 [14].

Two earlier studies in Bahrain examined the epidemiology of SARI across all age groups using national surveillance data. Both studies documented that influenza A was the most common viral pathogen, followed by SARS-CoV-2 and RSV. Additionally, patients aged 50 years or older, as well as those with chronic kidney, lung, or heart disease, experienced poorer outcomes [5,6]. However, these studies had limitations, including incomplete clinical information, potential diagnostic errors, and underreporting in surveillance systems. So far, no study in Bahrain has thoroughly examined viral respiratory infections in hospitalized adults using detailed clinical data.

A Bahrain-specific clinical dataset focused exclusively on adults is urgently needed because national decision-making currently relies on surveillance summaries that under-capture influenza and RSV cases and lack essential clinical detail, limiting the ability to design targeted vaccination strategies, optimize antiviral use, and anticipate seasonal surges that strain hospital capacity. A detailed understanding of the epidemiology and clinical impact of respiratory viruses is particularly important in Bahrain's diverse adult population, which includes many expatriate workers with varied health backgrounds and inconsistent access to preventive care. Identifying high-risk groups helps hospitals implement more effective diagnostic protocols and prepare for periods when viruses spread more easily. To our knowledge, this is the first study in Bahrain to analyze adult respiratory viral infections using detailed clinical data rather than surveillance summaries. Hence, this study aimed to thoroughly assess the burden, testing patterns, and clinical outcomes of these viruses among adults admitted with ARI at SMC during the 2023-2024 winter season.

## Materials and methods

Study design and setting

This retrospective cohort study included all adults admitted to the respiratory unit at SMC with ARI during the 2023-2024 winter season. SMC is the largest government hospital in Bahrain, with a capacity of approximately 1,100 beds and serving as the primary referral center for acute and chronic respiratory conditions. The study period spanned October 2023 to February 2024, corresponding to the peak seasonal circulation of respiratory viruses in the region.

Study population

Electronic medical records (EMRs) were reviewed to extract demographic, clinical, and laboratory information. Patients with repeated admissions during the study period were counted as separate episodes only if the admission diagnoses differed; repeated admissions for the same diagnosis were excluded to avoid duplication. The study included immunocompromised patients, reflecting the real-world clinical population admitted to the respiratory unit.

Testing policy and potential bias

Testing for respiratory viruses was clinician-driven and not standardized across all admissions. The decision to request SARS-CoV-2, influenza, or RSV testing was based on the treating team's clinical judgment. Middle East respiratory syndrome coronavirus (MERS-CoV) was not included because MERS testing is not part of routine diagnostic evaluation for ARI at SMC, and no suspected MERS cases were reported during the study period. Testing for SARS-CoV-2 and influenza was performed using rapid antigen detection or polymerase chain reaction (PCR), while RSV testing was performed only by PCR.

Inclusion criteria

Although the study focused on adults, the respiratory unit at SMC routinely admits patients aged ≥14 years, as per hospital policy, because adolescents in this age group are managed in adult medical wards rather than pediatric services. For this reason, patients aged 14-17 years were included to accurately reflect the real‑world clinical population admitted with ARI. Only 16 of the patients (1.7%) were aged 14-17 years, and their inclusion did not materially affect the overall findings. Eligibility criteria also require a documented clinical diagnosis of an ARI, including pneumonia, bronchial asthma exacerbation, COPD exacerbation, upper respiratory tract infection, or another acute respiratory condition as determined by the treating team. We included only patients whose EMRs had complete demographic, clinical, and outcome data to make sure the data were accurate and consistent.

Exclusion criteria

Patients were excluded only if they had repeated admissions for the same clinical diagnosis within a short interval that clearly represented continuation of the same illness episode rather than a new infection. If patients were admitted with the same diagnosis after enough time had passed, beyond the usual incubation period for respiratory viruses, these were counted as separate clinical episodes and included in the analysis. This method helped make sure that repeat or new infections were not accidentally left out. Admissions in which the primary reason for hospitalization was unrelated to ARI, or cases transferred to the respiratory unit for non‑respiratory conditions, were also excluded. Additionally, patients with incomplete or missing medical records that prevented the extraction of key variables were excluded. No further clinical or demographic exclusions were applied to preserve the real‑world nature of the cohort.

Data collection

Data were extracted from the EMRs by two trained researchers (two medical residents) using a standardized data collection form. To ensure accuracy and consistency, both reviewers independently cross‑checked a random sample of entries, and discrepancies were resolved through consensus with senior investigators (three respiratory consultants). The variables collected included patient demographics such as age, sex, and nationality, as well as pre-existing medical conditions, including diabetes, chronic lung disease, and cardiovascular disease. The dataset also included information on admission diagnosis, results of viral testing for SARS-CoV-2, influenza, and RSV using rapid multiplex PCR assays, and the use of antiviral medications such as oseltamivir and remdesivir. Clinical outcomes were documented, including ICU admission, the need for mechanical ventilation, length of hospital stay, and discharge status (alive or deceased). All data were initially entered into a customized Microsoft Excel (Microsoft® Corp., Redmond, WA) spreadsheet, where cleaning, deduplication, and accuracy checks were performed. Missing data were handled using a complete‑case approach; variables with unavailable or undocumented information were recorded as missing and were not imputed. This approach preserved the integrity of the dataset while reflecting real‑world clinical documentation practices. The finalized dataset was then imported into IBM SPSS Statistics for Windows (Version 26.0, IBM Corp., Armonk, NY) for statistical analysis.

Ethical statement

This study was approved by the Institutional Review Board of the Ministry of Health, Bahrain (approval no. 33-141224) on 14 December 2024. Informed consent was waived due to the study's retrospective design.

Statistical analysis

Descriptive statistics were used to summarize demographic and clinical characteristics. Categorical variables were presented as frequencies and percentages, while continuous variables were summarized using means and standard deviations or medians and ranges, depending on distribution. Associations between viral infection status (SARS-CoV-2, influenza, RSV) and demographic or clinical variables were assessed using Chi‑square tests or Fisher's exact tests, as appropriate. Only pre-infection variables, defined as characteristics present at the time of admission and prior to the availability of viral test results, were included in the adjusted model. These variables comprised age group, gender, diabetes, chronic lung disease, and pneumonia diagnosis. Importantly, pneumonia was treated as an initial admission diagnosis rather than a post‑infection outcome, ensuring that predictors reflected baseline clinical presentation and avoiding misclassification. A multivariate logistic regression model was employed to identify independent predictors of SARS-CoV-2 infection. All variables were coded according to standard clinical definitions documented in the EMR. Adjusted odds ratios (AORs) with 95% confidence intervals (CIs) were reported. Survival analysis was conducted to compare the length-of-stay (LOS) survival patterns between SARS-CoV-2-positive and SARS-CoV-2-negative patients. Kaplan-Meier curves were generated, and differences between groups were assessed using the log-rank (Mantel-Cox) test. Furthermore, in the Kaplan-Meier analysis, discharge was treated as the event of interest. Patients who died during hospitalization were censored at the time of death, as the analysis aimed to compare LOS patterns rather than mortality risk. To complete with Kaplan-Meier, a Cox proportional hazards regression model was performed to complement the Kaplan-Meier analysis and adjust for baseline differences. LOS (days) was used as the time variable, with discharge alive coded as the event, and in-hospital deaths censored. The main exposure was SARS-CoV-2 infection status. Covariates included age group, gender, diabetes mellitus, and chronic lung disease at admission. Hazard ratios (HRs) with 95% CIs were reported. The proportional hazards assumption was assessed using log-minus-log plots and was not violated. A p-value < 0.05 was considered statistically significant for all analyses. All statistical analyses were performed using IBM SPSS Statistics for Windows (Version 26.0, IBM Corp., Armonk, NY).

## Results

Demographic characteristics

**Figure 1:**
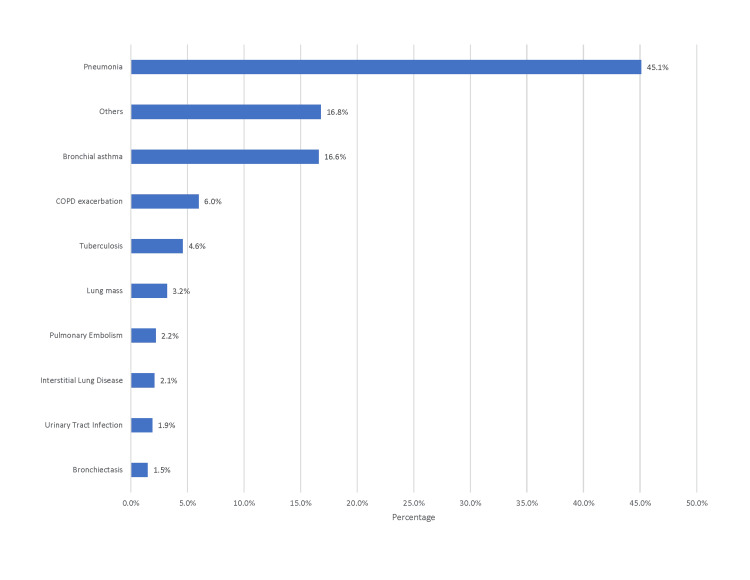
Admission diagnosis Image was created using Microsoft Word (Microsoft® Corp., Redmond, WA).

A total of 943 adults were admitted to the respiratory unit at SMC between October 2023 and February 2024. Table 1 presents the demographic and clinical characteristics of the patients. The largest proportion of patients was in the oldest age group (>70 years, N = 276; 29.3%), while the youngest group (14-30 years) accounted for the smallest share (N = 84; 8.9%). Gender distribution was nearly equal between males and females (N = 471; 49.9% vs. N = 472; 50.1%). Nationality distribution revealed that non-Bahraini patients comprised the majority (N = 681; 72.2%), substantially exceeding the proportion of Bahraini nationals. The most frequent length of hospital stay was three days or fewer (N = 314; 33.3%), whereas admissions exceeding 20 days were uncommon (N = 67; 7.1%). Among viral tests, SARS-CoV-2 screening was most common (N = 625; 66.3%), while RSV testing was least frequent (N = 57; 6.0%). Diabetes mellitus (N = 397; 42.1%) and chronic lung disease (N = 391; 41.5%) were the most common comorbidities, while solid organ transplant (0.1%) was rare. Pneumonia was the most frequent admission diagnosis (N = 425; 45.1%), followed by exacerbations of bronchial asthma (N = 157; 16.6%) and COPD (N = 57; 6%) (see Figure 1). Overall, 83 patients (8.8%) died during hospitalization, and 19 (2.0%) required ICU admission.

**Table 1:** Demographic and clinical characteristics of the patients (n = 943) Results are expressed as numbers and percentages, N (%).

Study variable	N (%)
Age group	
14-30 years	84 (8.9%)
31-40 years	106 (11.3%)
41-50 years	118 (12.5%)
51-60 years	153 (16.2%)
61-70 years	206 (21.8%)
>70 years	276 (29.3%)
Gender	
Male	471 (49.9%)
Female	472 (50.1%)
Nationality	
Bahraini	262 (27.8%)
Non-Bahraini	681 (72.2%)
Length of hospital stay in days	
≤3 days	314 (33.3%)
4-6 days	269 (28.5%)
7-10 days	158 (16.8%)
11-20 days	135 (14.3%)
>20 days	67 (7.1%)
Discharge status	
Died	83 (8.8%)
Alive	860 (91.2%)
Requiring ICU admission	
No	924 (98.0%)
Yes	19 (2.0%)
SARS-CoV-2 infection	
Not tested	318 (33.7%)
Tested	625 (66.3%)
Influenza infection	
Not tested	781 (82.8%)
Tested	162 (17.2%)
RSV infection	
Not tested	886 (94.0%)
Tested	57 (6.0%)

Respiratory virus testing patterns

Of the 943 admitted patients, 625 (66%) underwent testing for respiratory viruses. SARS-CoV-2 testing was performed for all 625 tested patients (100%). In contrast, influenza testing was performed in only 162 patients (17.2% of the total cohort; 26% of those tested), and RSV testing in only 57 patients (6% of the total cohort; 9.1% of those tested). As noted in the dataset, "overall RSV and influenza testing were under-requested, and this affects positivity rates." Testing rates varied by admission diagnosis. Testing was most frequent among patients with pneumonia (N = 308; 72%), COPD exacerbation (N = 43; 75%), bronchial asthma exacerbation (N = 92; 60%), severe upper respiratory tract infection (N = 105; 94%), and other miscellaneous diagnoses (N = 105; 94%). Testing was less frequent among patients with bronchiectasis (N = 6; 43%) and tuberculosis (N = 13; 30%) (Table 2).

**Table 2:** Respiratory virus testing among the study population (N = 943) Results are expressed as numbers and percentages, N (%). COPD, chronic obstructive pulmonary disease; ILD, interstitial lung disease; PE, pulmonary embolism; TB, tuberculosis; URTI, upper respiratory tract infection

Diagnosis	Total patients N (%)	COVID testing		Influenza testing		RSV testing	
		Done N (%)	Not done N (%)	Done N (%)	Not done N (%)	Done N (%)	Not done N (%)
Pneumonia	425 (45.1%)	308 (72.5%)	117 (27.5%)	74 (17.4%)	351 (82.6%)	28 (6.6%)	397 (93.4%)
Bronchial asthma exacerbation	157 (16.6%)	92 (58.6%)	65 (41.4%)	31 (19.7%)	126 (80.3%)	9 (5.7%)	148 (94.3%)
COPD exacerbation	57 (6.0%)	43 (75.4%)	14 (24.6%)	10 (17.5%)	47 (82.5%)	3 (5.3%)	54 (94.7%)
Bronchiectasis	14 (1.5%)	6 (42.9%)	8 (57.1%)	0 (0.0%)	14 (100%)	0 (0.0%)	14 (100%)
URTI	18 (1.9%)	14 (77.8%)	4 (22.2%)	3 (16.7%)	15 (83.3%)	2 (11.1%)	16 (88.9%)
Lung mass	30 (3.2%)	13 (43.3%)	17 (56.7%)	8 (26.7%)	22 (73.3%)	3 (10.0%)	27 (90.0%)
TB	43 (4.6%)	13 (30.2%)	30 (69.8%)	3 (7.0%)	40 (93.0%)	0 (0.0%)	43 (100%)
PE	21 (2.2%)	19 (90.5%)	2 (9.5%)	3 (14.3%)	18 (85.7%)	1 (4.8%)	20 (95.2%)
ILD	20 (2.1%)	12 (60.0%)	8 (40.0%)	5 (25.0%)	15 (75.0%)	2 (10.0%)	18 (90.0%)
Other diagnosis	158 (16.8%)	105 (66.5%)	53 (33.5%)	25 (15.8%)	133 (84.2%)	9 (5.7%)	149 (94.3%)
Total patients	943 (100%)	625 (66.3%)	318 (33.7%)	162 (17.2%)	781 (82.8%)	57 (6.0%)	886 (94.0%)

RSV infection

Among the 57 patients tested for RSV, three were positive (5.2%). There were no statistically significant differences between RSV-positive and RSV-negative patients in age, gender, nationality, comorbidities, or clinical outcomes (p > 0.05). Two out of three RSV-positive patients died (66.7). None of the RSV‑positive patients had received RSV vaccination, which was not yet part of the national immunization program, and no patient received RSV-specific antiviral therapy. Given the limited number of cases, RSV-related findings should be interpreted with caution due to insufficient statistical power.

SARS-CoV-2 infection

Among the 625 patients tested for SARS-CoV-2, 32 were positive (5.1%). SARS-CoV-2 positivity was significantly associated with female gender (N = 23; 71.9%, p = 0.008), age >60 years (N = 22; 68.8%, p = 0.045), and diabetes mellitus (N = 21; 65.6%, p = 0.009). Mortality was significantly higher among SARS-CoV-2-positive patients compared with negative patients (N = 7; 21.9% vs. N = 60; 10.1%; p = 0.036) (Table 3).

**Table 3:** Relationship between SARS-CoV-2 infection and the demographic and clinical characteristics of the patients (n = 625) * Results are expressed as numbers and percentages, N (%). ICU, intensive care unit; NA, not applicable ^*^ Not tested cases were excluded from the analysis. ^†^ Some patients have multiple comorbidities. ^§^ p-value has been calculated using the chi-square test. ^‡^ Fisher’s exact test was used for cells with expected counts <5. ^**^ Significant at p < 0.05 level.

Factor	SARS-CoV-2 infection		χ²	p-value ^§^
	Negative N (%) (n = 593)	Positive N (%) (n = 32)		
Age group				
≤60 years	293 (49.4%)	10 (31.3%)	4.009	0.045 **
>60 years	300 (50.6%)	22 (68.8%)		
Gender				
Male	310 (52.3%)	09 (28.1%)	7.087	0.008 **
Female	283 (47.7%)	23 (71.9%)		
Nationality				
Bahraini	169 (28.5%)	08 (25.0%)	0.183	0.669
Non-Bahraini	424 (71.5%)	24 (75.0%)		
Length of hospital stay				
≤7 days	341 (57.5%)	22 (68.8%)	1.577	0.209
>7 days	252 (42.5%)	10 (31.3%)		
Discharge status				
Died	60 (10.1%)	07 (21.9%)	4.385	0.036 **
Alive	533 (89.9%)	25 (78.1%)		
Requiring ICU admission				
No	574 (96.8%)	32 (100%)	NA	0.616 ^‡^
Yes	19 (3.2%)	0		
Influenza infection				
Negative	140 (93.3%)	12 (100%)	NA	1.000 ^‡^
Positive	10 (6.7%)	0		
Most common comorbidities ^†^				
Diabetes	250 (42.2%)	21 (65.6%)	6.808	0.009 **
Chronic lung disease	235 (39.6%)	10 (31.3%)	0.894	0.344
Cardiovascular disease	170 (28.7%)	13 (40.6%)	2.096	0.148
Diagnosed with pneumonia				
No	305 (51.4%)	12 (37.5%)	2.358	0.125
Yes	288 (48.6%)	20 (62.5%)		
Diagnosed with bronchial asthma				
No	503 (84.8%)	30 (93.8%)	NA	0.206 ^‡^
Yes	90 (15.2%)	02 (6.3%)		

In a multivariate logistic regression analysis (Table 4), we identified several independent predictors of SARS-CoV infection. Women exhibited nearly threefold higher odds of SARS-CoV-2 infection compared to men (AOR = 2.78, 95% CI: 1.25-6.20, p = 0.012). Diabetes was another key factor. Patients with diabetes had more than twice the odds of infection (AOR = 2.54, 95% CI: 1.13-5.70, p = 0.024), supporting the hypothesis that metabolic conditions increase risk during respiratory outbreaks. Patients aged over 60 years showed a trend toward lower odds of infection (AOR = 0.46, 95% CI: 0.20-1.04), although this finding was not statistically significant after adjustment to a regression model (p = 0.062). Pneumonia exhibited a non-significant trend toward increased odds of SARS-CoV-2 infection (AOR 1.874, p = 0.098), indicating that patients with pneumonia were nearly twice as likely to test positive; however, the evidence was insufficient to establish a definitive association. Conversely, chronic lung disease was associated with a non-significant reduction in odds (AOR 0.657, p = 0.291), suggesting that pre-existing respiratory disease did not substantially affect SARS-CoV-2 positivity within this cohort. Overall, findings suggest that female gender and diabetes emerged as the significant independent predictors of SARS-CoV-2 infection in our population group.

**Table 4:** Multivariate logistic regression analysis to determine the predictors of SARS-CoV-2 infection (n = 625) * Ref, reference category. ^*^ Not tested cases were excluded from the analysis. ^**^ Significant at p < 0.05 level. Adjusted for nationality, length of hospital stay, and cardiovascular disease.

Factor	AOR	95% CI	p-value
Age group			
≤60 years	Ref	-	-
>60 years	0.460	0.203-1.041	0.062
Gender			
Male	Ref	-	-
Female	2.781	1.248-6.197	0.012 **
Diagnosed with pneumonia			
No	Ref	-	-
Yes	1.874	0.890-3.944	0.098
Diabetes			
No	Ref	-	-
Yes	2.540	1.132-5.699	0.024 **
Chronic lung disease			
No	Ref	-	-
Yes	0.657	0.301-1.432	0.291

**Figure 2:**
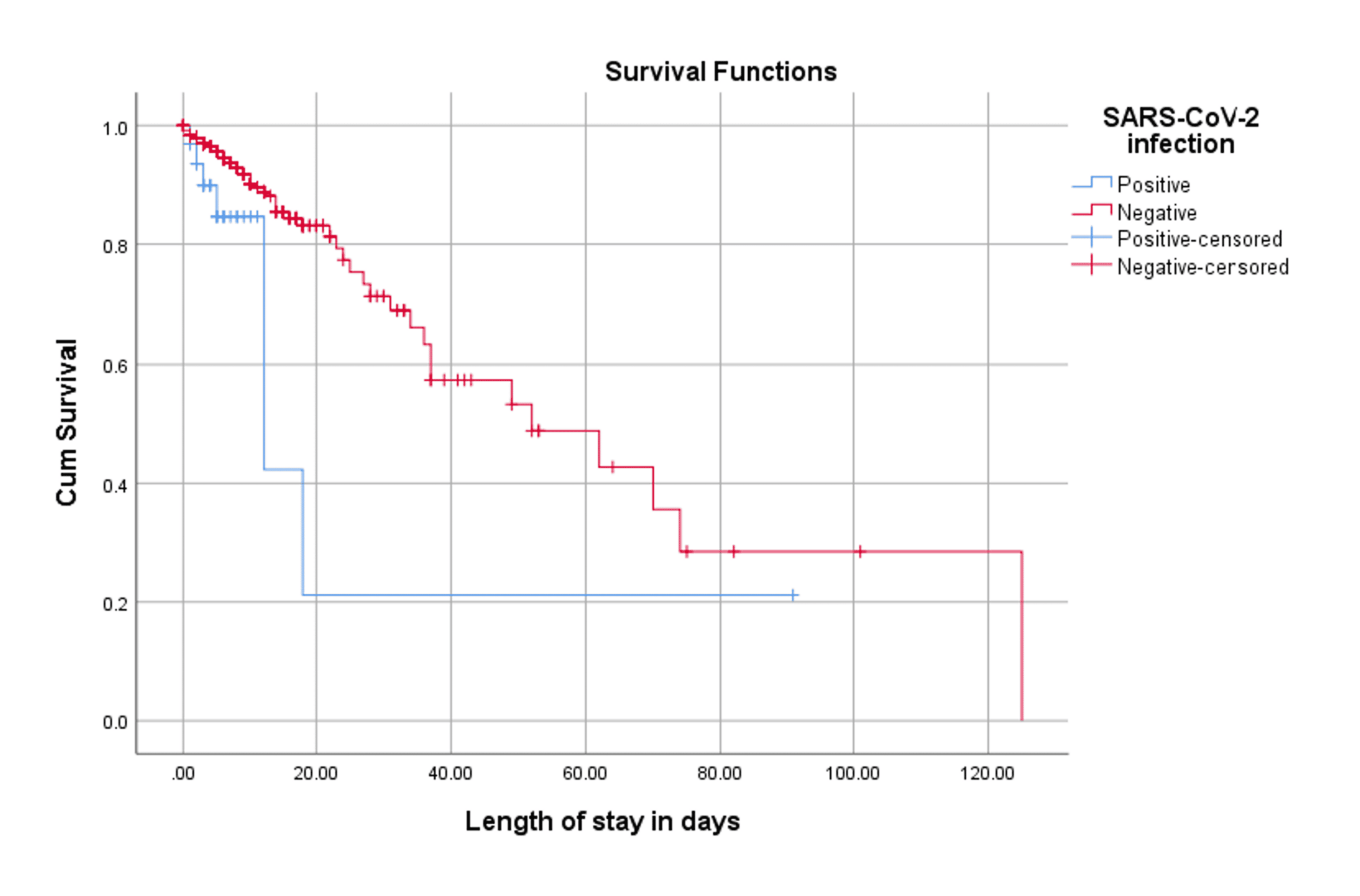
Survival plot of SARS-CoV-2 patients in relation to length of hospital stay in days Image was generated using IBM SPSS Statistics for Windows (Version 26.0, IBM Corp., Armonk, NY).

Figure 2 presents the survival plot patterns of patients with SARS-CoV-2. Kaplan-Meier curves demonstrated that SARS-CoV-2-positive patients had significantly shorter LOS survival compared with SARS‑CoV-2-negative patients. The mean time to discharge was 28.6 days for SARS-CoV-2-positive patients vs. 63.4 days for SARS-CoV-2-negative patients (χ² = 5.786; log-rank p = 0.016). This indicates that SARS-CoV-2 infection was associated with earlier termination of hospitalization - either through discharge or death - reflecting a more severe clinical trajectory.

A Cox proportional hazards regression model was performed in Table 5 to further evaluate whether SARS-CoV-2 infection independently predicted time to discharge after adjusting for baseline characteristics. Based on the results, SARS-CoV-2 infection was independently associated with a significantly faster time to discharge. Patients who tested positive had a 2.35-fold higher hazard of discharge compared with SARS-CoV-2-negative patients (HR = 2.348, 95% CI: 1.005-5.489, p = 0.049). This finding indicates that SARS-CoV-2-positive patients were discharged more rapidly than their negative counterparts, even after accounting for baseline imbalances.

**Table 5:** Cox proportional hazards regression model for predictors of time to discharge among SARS-CoV-2-tested patients (n = 625) * ^* ^Not tested cases were excluded from the analysis. ^** ^Significant at p < 0.05 level. Adjusted for age group, gender, diabetes mellitus, and chronic lung disease.

Variable	B	SE	Wald	df	p-value	Hazard ratio (exp(B))
SARS-CoV-2 infection (positive vs. negative)	0.854	0.433	3.884	1	0.049**	2.348

Influenza infection

A total of 162 patients were tested for influenza; 10 were positive for a positivity rate of 6%. There was no significant difference in age, gender, or nationality as risk factors for influenza acquisition (p > 0.05). The main risk factors for influenza infection were underlying chronic lung disease and bronchial asthma (p = 0.045 and 0.023, respectively). There were no significant differences in mortality, ICU admission, or length of stay between influenza‑positive and influenza-negative patients (p > 0.05). Among the 10 positive influenza cases, there was no mortality and no ICU admissions, despite none having received the annual influenza vaccine in the preceding year and only 5 (50%) receiving oseltamivir during the hospital stay (Table 6).

**Table 6:** Relationship between influenza infection and the demographic and clinical characteristics of the patients with influenza infection (N = 162) * Results are expressed as numbers and percentages, N (%). ^*^ Not tested cases were excluded from the analysis. ^†^ Some patients have multiple comorbidities. ^§^ Fisher’s exact test was used for cells with expected counts <5. ^**^ Significant at p < 0.05 level.

Factor	Influenza infection		p-value ^§^
	Negative N (%) (n = 152)	Positive N (%) (n = 10)	
Age group			
≤60 years	73 (48.0%)	06 (60.0%)	0.527
>60 years	79 (52.0%)	04 (40.0%)	
Gender			
Male	81 (53.3%)	04 (40.0%)	0.520
Female	71 (46.7%)	06 (60.0%)	
Nationality			
Bahraini	47 (30.9%)	02 (20.0%)	0.726
Non-Bahraini	105 (69.1%)	08 (80.0%)	
Length of hospital stay			
≤7 days	83 (54.6%)	07 (70.0%)	0.514
>7 days	69 (45.4%)	03 (30.0%)	
Discharge status			
Died	14 (09.2%)	0	0.603
Alive	138 (90.8%)	10 (100%)	
Requiring ICU admission			
No	151 (99.3%)	10 (100%)	1.000
Yes	01 (0.70%)	0	
Most common comorbidities ^†^			
Diabetes	62 (40.8%)	04 (40.0%)	1.000
Chronic lung disease	67 (44.1%)	08 (80.0%)	0.045 **
Cardiovascular disease	44 (28.9%)	02 (20.0%)	0.726
Diagnosed with pneumonia			
No	81 (53.3%)	07 (70.0%)	0.347
Yes	71 (46.7%)	03 (30.0%)	
Diagnosed with bronchial asthma			
No	126 (82.9%)	05 (50.0%)	0.023 **
Yes	26 (17.1%)	05 (50.0%)	

Factors associated with mortality among SARS-CoV-2 positive patients

In patients who tested positive for SARS-CoV-2, most demographic and clinical factors, such as age group, gender, nationality, and COVID-19 vaccination status, did not show a significant association with mortality (all p > 0.05). Although mortality was higher among patients aged over 60 years (N = 6; 85.7% vs. N = 1; 14.3%), this difference did not reach statistical significance (p = 0.387), possibly due to the limited sample size. Gender and nationality also did not show a meaningful association with mortality (p > 0.05). The only factor that showed a statistically significant association with mortality was antiviral therapy (p = 0.043). Patients who died were significantly less likely to receive antiviral treatment (N = 3; 42.9% vs. N=24; 96%). This pattern likely reflects clinical decision‑making, where antivirals were administered early in patients who were stable enough to receive treatment, while those who deteriorated rapidly may not have been eligible or may have presented too late for antiviral initiation. COVID-19 vaccination status did not significantly influence mortality (p = 0.353), although unvaccinated patients showed a higher proportional mortality rate (Table 7).

**Table 7:** Factors that influence mortality among SARS-CoV-2 positive cases (n = 32) Results are expressed as numbers and percentages, N (%). ^§^ Fisher’s exact test was used for cells with expected counts <5. ^**^ Significant at p < 0.05 level.

Factors	SARS-CoV-positive		p-value ^§^
	Dead (n = 07)	Alive (n = 25)	
Age group			
≤60 years	1 (14.3%)	9 (36.0%)	0.387
>60 years	6 (85.7%)	16 (64.0%)	
Gender			
Male	1 (14.3%)	8 (32.0%)	0.640
Female	6 (85.7%)	17 (68.0%)	
Nationality			
Bahraini	0	8 (32.0%)	0.150
Non-Bahraini	7 (100%)	17 (68.0%)	
Use of antiviral			
Yes	3 (42.9%)	24 (96.0%)	0.043 **
No	4 (57.1%)	1 (4.0%)	
COVID vaccination			
Yes	6 (85.7%)	24 (96.0%)	0.353
No	1 (14.3%)	1 (4.0%)	

## Discussion

This study provides one of the most comprehensive clinical assessments of viral respiratory infections among hospitalized adults in Bahrain. This study used detailed EMRs rather than surveillance summaries to examine the burden, testing patterns, and outcomes of SARS-CoV-2, influenza, and RSV during the 2023-2024 winter season. While patient-level clinical data provide richer clinical detail than surveillance summaries, the findings should be interpreted with caution because viral testing at SMC was clinician-driven rather than standardized. This introduces selection bias, as patients who appeared more severely ill or clinically suspicious were more likely to be tested. The findings show significant testing gaps, distinct risk profiles for each virus, and a need to improve diagnostic and preventive strategies for high-risk adults.

Month-wise patterns of infection showed that SARS-CoV-2 activity peaked in December and January, coinciding with the mid-winter increase in respiratory admissions. Influenza cases occurred mainly from November to January, reflecting Bahrain’s typical seasonal influenza wave. RSV activity remained low, with only sporadic detections in November and early December and no sustained circulation. These trends are consistent with regional patterns and can support future surveillance and preparedness planning.

Burden and predictors of SARS‑CoV-2 infection

Despite a SARS-CoV-2 positivity rate of only 32 (5.1%), the clinical impact was severe. Infections were closely associated with older age, diabetes, and an increase in in-hospital mortality. These results align with global and regional evidence that older adults and individuals with chronic diseases remain at higher risk for severe COVID-19, even with widespread immunity and vaccination [1,5,6]. The link with diabetes supports existing knowledge that metabolic dysfunction weakens antiviral immunity and increases inflammation [8].

The higher SARS-CoV-2 positivity among women in our cohort contrasts with international trends, where men more commonly experience severe disease. This pattern may reflect underlying social or exposure-related factors in the population; however, because occupation was not captured in our dataset, these interpretations remain speculative and should be viewed with caution. These contextual factors likely explain the gender distribution more than biological susceptibility, although further investigation is warranted.

Age and diabetes remained significant predictors of SARS-CoV-2 infection in multivariate analysis, consistent with evidence that immune aging and metabolic disease increase vulnerability to viral respiratory infections [15-17]. A prior Bahraini ICU study similarly demonstrated that age ≥60 years was the strongest independent predictor of mortality among hospitalized COVID-19 patients [18], reinforcing the central role of immune senescence and chronic disease in determining clinical severity.

Furthermore, the fact that most SARS-CoV-2 cases in Bahrain are among non-Bahraini patients has significant social and public health effects. Many expatriate workers live in crowded housing and work in jobs that require close contact with others, which can raise their risk of catching respiratory viruses. Non-Bahraini residents may also have trouble getting healthcare early, whether because they are not aware of available services, face financial challenges, or delay seeking care due to work demands. These social and economic factors may potentially influence infection risk, but because socioeconomic data were not collected in this study, these interpretations remain suggestive rather than conclusive. Recognizing these differences is important for creating targeted public health programs, making preventive services more accessible, and improving monitoring for at-risk groups.

SARS-CoV-2 infection was also associated with markedly higher mortality (21.9% vs. 10.1%) and significantly shorter survival. This association warrants cautious interpretation, as viral testing was conducted according to clinical judgment rather than standardized criteria. Consequently, clinicians may have preferentially tested patients presenting with more severe illness, potentially resulting in a higher proportion of critically unwell individuals among those who tested positive for SARS-CoV-2. This selection bias may partially account for the increased mortality and reduced survival observed in this group. This aligns with international studies showing persistent morbidity among older adults and those with comorbidities in the post‑pandemic period [19]. Only 12.5% of SARS-CoV-2-positive patients received remdesivir, and the observed association between antiviral use and mortality likely reflects confounding by indication, as antivirals were typically initiated after clinical deterioration rather than indicating treatment-related harm.

Diabetes is considered a risk factor because it causes changes in the renin-angiotensin system (RAS), leads to chronic inflammation, and increases oxidative stress. Together, these problems weaken antiviral defenses and make clinical outcomes worse [20]. These factors help explain why diabetes was a strong predictor of SARS-CoV-2 infection and poor outcomes in our group.

Influenza infection

Influenza positivity was 10 (6%), but only 162 (17%) of the cohort were tested, suggesting substantial under-ascertainment. Influenza-positive patients in this study experienced favorable outcomes, with no ICU admissions or deaths. This finding contrasts with global literature, which documents significant influenza-related morbidity among adults with chronic lung disease, asthma, and cardiovascular disease [2,3,8]. The mild outcomes observed may be attributable to the small sample size, early clinical presentation, or the circulation of less virulent influenza strains during the study period. Importantly, none of the influenza-positive patients had received the annual influenza vaccine, emphasizing the need to improve adult vaccination uptake in Bahrain. The significant association between influenza infection and chronic lung disease or asthma identifies a clear target group for enhanced vaccination efforts and early antiviral therapy. Influenza infection was significantly associated with chronic lung disease and bronchial asthma, consistent with established literature showing that influenza exacerbates underlying airway disease [8]. None of the influenza-positive patients died or required ICU admission, although this should be interpreted cautiously given the small sample size and selective testing.

RSV infection

RSV testing was extremely limited, with only 57 patients tested, of whom three were positive. Although two of the three RSV-positive patients died, this finding was not statistically significant due to the small sample size. Given the very small number of RSV-positive cases and the presence of substantial baseline comorbidities, these deaths cannot be definitively attributed to RSV-specific virulence and should be interpreted with caution. RSV is an important respiratory pathogen across all age groups, causing substantial morbidity not only in older adults and individuals with chronic lung or heart disease but also in children. Recent evidence from Gulf settings with similar climate and demographic profiles, including Saudi Arabia, has also documented clear seasonal RSV circulation and clinically significant illness [21]. Hospitalization rates can reach 136 to 255 per 100,000 adults aged 65 and older [9,10], and ICU admission rates range from 10% to 31% [12]. A recent multicenter study found that RSV is linked to higher ICU admission and mortality risks than influenza or COVID-19 in older adults [14]. None of the RSV-positive patients had been vaccinated, since RSV vaccines were not yet included in the national immunization program during the study period.

Antiviral therapy for respiratory viral pathogens

Evidence demonstrates that early initiation of antiviral therapy for influenza and SARS-CoV-2 reduces mortality and shortens hospital length of stay [22-25]. Despite this, antiviral use in our cohort was limited. Only 50% of influenza‑positive patients received oseltamivir, and just 12.5% of SARS-CoV-2-positive patients received remdesivir. Mortality was lower among patients who received antivirals, although this association likely reflects confounding by clinical eligibility and timing rather than a direct treatment effect.

Vaccination for respiratory viral pathogens

Among SARS-CoV-2-positive patients, 93% had received three vaccine doses during 2021-2022. In contrast, none of the influenza-positive patients had received the annual influenza vaccine, despite 70% being ≥50 years and eligible under Bahrain's national immunization schedule [26]. Previous Bahraini studies similarly reported very low influenza vaccine uptake [6]. The extremely low vaccination rate among ICU COVID-19 patients in the Janahi et al. study (97.8% unvaccinated) further highlights the protective effect of vaccination and the need for sustained public health efforts [18].

Mortality among patients with respiratory viral infections

Although RSV infection demonstrated the highest mortality proportion in this cohort (N = 2; 66.7%), this figure cannot be interpreted as a true mortality estimate due to the limited number of tested patients and the critical condition of two individuals at baseline. The very small sample size and selective testing likely resulted in an inflated observed mortality rate. SARS-CoV-2 (N = 7; 21.9%) was recognized as the secondary infection, with no deaths among influenza-positive patients. Although the RSV mortality rate observed here was higher than previously reported, this likely reflects selective testing of more critically ill patients. Prior studies consistently demonstrate the substantial severity of RSV in older adults. Ackerson et al. reported that, among hospitalized older adults, RSV was associated with higher rates of ICU admission, mechanical ventilation, and mortality than influenza, underscoring its substantial clinical impact [27]. In a large U.S. study of 5,784 hospitalized adults aged 60 and older, RSV-related deaths were 1.39 times higher than those from SARS-CoV-2 and 2.08 times higher than from influenza [14]. These results underscore the importance of improved RSV testing, early detection of severe RSV cases, and targeted prevention efforts.

The higher mortality rate and shorter time-to-discharge observed among SARS-CoV-2-positive patients in this study likely reflect a high-acuity subset selected through discretionary testing rather than the broader adult population of Bahrain. Because 72.2% of the cohort were non-Bahraini expatriates, whose health-seeking behavior, occupational exposures, and access pathways differ from Bahraini nationals, the findings should be interpreted as representative of the hospitalized population presenting to SMC rather than the national demographic structure. Interpretation of the Kaplan-Meier curves also warrants caution. Treating death as a censored observation in a discharge-based survival model introduces a competing-risk limitation, as mortality directly prevents the event of interest and may bias estimates of the clinical trajectory. Importantly, the Cox proportional hazards regression model confirmed that the shorter time-to-discharge observed in the Kaplan-Meier analysis remained significant after adjustment, demonstrating that the effect was not solely attributable to age, gender, or comorbidity differences between groups.

Testing patterns and structural bias

One important finding is that viral testing is often inconsistent and underused. SARS-CoV-2 testing was done more regularly, but tests for influenza and RSV were much less common, especially in patients with pneumonia, bronchiectasis, and tuberculosis. This selective testing can cause bias, underestimate true positivity rates, and make it hard to compare the prevalence of different viruses. Similar problems have been found in Bahrain’s national SARI surveillance system, which has also reported too little testing and incomplete clinical data [5,6]. Using standardized three-virus testing panels for adults with ARI could improve diagnosis, guide antiviral treatment, and strengthen national surveillance.

Strengths of the study

A key strength of this study is its large sample size of 943 adult patients admitted with ARI in one winter season. This gives a strong basis for understanding how viral respiratory infections affect people in Bahrain. Detailed demographic, clinical, and outcome data were collected from EMRs. This approach enabled more accurate identification of risk factors and disease severity compared to studies relying solely on surveillance data. We performed a survival analysis to compare outcomes in patients with SARS-CoV-2 infection and found clear differences in survival rates between infected and uninfected patients. This research was conducted at SMC, the main secondary and tertiary care hospital in Bahrain. SMC serves people of all ages from every governorate. Since Bahrain has a population of about 1.64 million, the patients at SMC represent the country's demographics. This makes our findings relevant to the wider adult population. Additionally, a small number of adolescents aged 14-17 years were included, reflecting SMC’s admission policy in which patients ≥14 years are managed in adult wards. Their inclusion preserves the real-world nature of the cohort and represents a very small proportion of the sample.

Study limitations

This study has several important limitations. First, the low testing rates for influenza (N = 162; 17.2%) and RSV (N = 57; 6%) substantially constrain the interpretation of viral prevalence. Because testing was performed at the discretion of clinicians rather than through a standardized protocol, the tested population may not represent the full clinical spectrum of admitted patients. This introduces potential selection bias, as clinicians may have preferentially tested individuals with more severe symptoms, atypical presentations, or perceived higher risk. Consequently, the observed positivity rates likely underestimate the true burden of influenza and RSV in this cohort. Second, the small number of confirmed cases limited the ability to detect meaningful associations between viral infections and clinical outcomes. Findings related to RSV, in particular, should be interpreted as descriptive rather than inferential. These limitations underscore the need for standardized, multi‑pathogen testing protocols to strengthen surveillance, reduce diagnostic variability, and enable more reliable comparisons across respiratory pathogens. Third, the Kaplan-Meier analysis censored patients who died during hospitalization because the outcome of interest was time-to-discharge rather than mortality. However, censoring deaths may introduce selection bias, as patients with severe illness who died had shorter lengths of stay for reasons unrelated to discharge. This may have influenced the observed differences in LOS survival between SARS-CoV-2-positive and SARS-CoV-2-negative patients. Finally, although this study was conducted in a single hospital, SMC functions as the main public referral center for ARI and receives patients from all governorates. This broad catchment enhances the relevance of the findings, but generalizability remains limited because the cohort reflects individuals who seek care at SMC rather than the full national demographic distribution.

Recommendations

Although this study has limitations, it provides important insights into how respiratory viral infections affect adults in Bahrain. These findings highlight the need for standardized testing criteria and clearer case definitions to reduce selection bias and ensure consistent identification of respiratory viral infections. Strengthening such protocols, alongside increasing clinician awareness, would help ensure that patients with SARI are tested more systematically for influenza, RSV, and SARS-CoV-2. This study also highlights the importance of early antiviral treatment for high-risk adults and underscores the need to strengthen vaccination uptake for influenza, RSV, and COVID-19 among older adults and individuals with chronic conditions, including both Bahraini nationals and expatriate residents. Implementing routine three-virus testing panels during peak respiratory seasons would not only improve case detection but also support more timely antiviral initiation, guide infection control measures, and generate more robust epidemiologic data for national decision-making. Furthermore, given that female gender and diabetes emerged as independent predictors of SARS-CoV-2 infection in this cohort, targeted preventive strategies are warranted. For diabetic patients, enhanced vaccination uptake, early testing during respiratory illness, and prompt antiviral initiation may reduce the risk of severe outcomes. The higher positivity among women, likely reflecting occupational and exposure‑related factors rather than biological susceptibility, highlights the need for focused health education and improved access to testing for women working in caregiving, domestic, and service roles. Integrating these high-risk groups into national vaccination and screening campaigns may improve early detection and reduce disease burden.

## Conclusions

This study addresses a significant evidence gap by presenting adult-specific clinical data on SARS-CoV-2, influenza, and RSV infections in Bahrain. Although SARS-CoV-2 infection was relatively uncommon, it was associated with markedly worse clinical outcomes, including higher in-hospital mortality and shorter survival, particularly among older adults and individuals with diabetes. Influenza and RSV were notably under-tested, limiting the accuracy of burden estimates and underscoring the need for standardized diagnostic protocols. The results underscore the importance of consistent three-virus testing, prompt initiation of antiviral therapy, and targeted vaccination strategies to enhance protection for high-risk adults and improve Bahrain's preparedness for seasonal respiratory virus surges.
