# Correction: Burden and Outcomes of Viral Respiratory Infections Among Adults in Bahrain: A Retrospective Cohort Study

**DOI:** 10.7759/cureus.c428

**Published:** 2026-05-08

**Authors:** Safa Alkhawaja, Rommel Acunin, Thuraya Zaid, Afaf Mohamed, Mohamed Alsaad, Mohamed Makhlooq, Muneer Mahdi, Alaa M Alzamrooni, Athraa S Naser, Nermin Saeed

**Affiliations:** 1 Medicine, Government Hospitals, Manama, BHR; 2 Infection Control, Government Hospitals, Manama, BHR; 3 Internal Medicine/Respiratory Unit, Government Hospitals, Manama, BHR; 4 Public Health, Ministry of Health - Bahrain, Manama, BHR; 5 Internal Medicine, Frimley Park Hospital, Frimley, GBR; 6 Internal Medicine, Arabian Gulf University, Manama, BHR; 7 Internal Medicine, Government Hospitals, Manama, BHR; 8 Pathology, Government Hospitals, Manama, BHR

This article has been corrected to include the following acknowledgement: The authors would like to acknowledge Mrs. Fadheela Alawi for her early involvement during the pilot phase of the project.

